# Supplementary material for: Organizing adult attachment in alternative ways: A qualitative assessment of schemas antithetical to the secure base script
Source: PLoS One. 2026 May 26;21(5):e0349710. doi: 10.1371/journal.pone.0349710 (PMC13210227; doi:10.1371/journal.pone.0349710)
Supplement: S1 File — (PDF) [file pone.0349710.s001.pdf]

S1: Mock Adult Attachment Interview- Role Reversal Alternative Schema

To increase the reader's understanding of the coding process, we provide this **mock, fictionalized** Adult Attachment Interview (Q1-Q6), with assigned codes for interview chunks describing potential alternative schemas.

***SUMMARY CODING**: Throughout the transcript, the relationship is most often described in terms of the child taking on the role of parent, with the parent receiving consistent emotional care and instrumental support from the child. Theme-like nature to the schema, Role Reversal being describe recurrently in questions 2,3 and 6.*

Q1. Could you tell me a little bit about your family when you were growing up and who would you say raised you?

Um, well, really it was just my mom who raised me. She and I were really like partners in crime and had each others backs for the most part **[*Coding: May be indicative of an AS (Enmeshed or maybe Role Reversal), but need more context*]**. My dad wasn't really involved in my life, my parents split when I was about 4 years old and I saw him very rarely after that, he moved to another state.

Q2. Now I'd like you to try to describe your relationships with your parents growing up. What was it like with your mom and with your dad between ages 5 and 12?

With my mom, I'd say it was… close, I always made sure I was there for her when she needed me when she was having a tough time.. I think I was the one kind of… keeping her going a lot of the time. She had a lot on her plate — working long hours, money was always tight — so I learned pretty young how to make dinner, even pay the bills, that kind of thing. I

felt proud of that, like I was… helping her stay on track [***Coding: May be indicative of an AS (Enmeshed or maybe Role Reversal), but need more context. Is the theme recurring further?***]. With my dad… he wasn't around much after the divorce, so it was just me and Mom for the most part. He wasn't really a parent to me to be honest.

Q3. Okay, thank you for describing that. Now, could you give me five adjectives that reflect your relationship with your mother during childhood, between the ages of 5 and 12? I'll write them down and we'll talk about what is it you remember that made you choose that specific word, maybe you have examples of specific events.

Okay... I'd say the first one is responsible...I always felt like I was the one holding things together [***Coding: AS Role Reversal- Holding things together]***. Next, I'd say protective. Then, trust maybe. I'd also say care is one. And last one.... Grown up.

Q3a. So, for first word that you used to describe that early relationship with your mom was responsible. Can you tell me a little bit more about that?

Yeah, so I remember that definitely things were responsible between us, she'd take care of me of course, put a roof over my head, get me my meals [***Coding: Mother is described providing instrumental care]***. And it was two-way street, I'd take care of her too. Help her remember stuff, listen to her problems, stuff like that. I was responsible early on with her ***[Coding: Child taking care of mother as well, both emotionally and instrumentally- Role Reversal]***.

Could you tell me a specific time when you felt the relationship with your mom was responsible?

Hmm.. I remember one time the boyfriend she had at the time, I was around 8, they had just broken up and she took it really hard. She didn't get out of bed for a few days. I was around 9, so I could already cook so I took on that role for those days. I could make eggs and coffee,

sandwiches, little things like that so I'd make myself something but also bring it to her room. I'd come home after school and check on her, listen to her and hold her while she was crying. We were like best friends, she could lean on me ***[Coding: AS Role Reversal- Offering support to mother, takes on parental role to offer emotional support and instrumental care].***

3b. Okay, that's a great example. Next, you chose the word loving to describe the relationship with your mom when you were between 5 and 12 years old. Can you tell me about that?

Like I said, we were like best friends, there was a lot of love between us. I was her daughter but I was also like her friend, so it was loving because I could help her just as much as she helped me as a mom. She worked really hard to make ends meet and give me a roof over my head, the least I could do was help her when she needed me ***[Coding: Identifies the relationship as a friendship, but idea that something is owed back because mother provides instrumental, basic care. AS Role Reversal]***.

Can you think of a specific example of the relationship being loving?

I guess in general we took care of each other. Like, when money was tight, I gave her my babysitting money or money from birthdays or little part-time jobs like distributing newspapers, to help tide things over. I think that was very loving between us [***Coding: AS Role Reversal]***.

3c. Okay, and the next word you used to describe the relationship with your mom was trust, can you tell me about that?

I think she put a lot of trust in me, more than other moms do with their kinds typically. She told me things I don't think most moms tell their kids. Looking back, I can see that maybe it wasn't always appropriate, but she would tell me a lot of things about my dad and their divorce, or about the breakups with her boyfriends throughout the years [***Coding: AS Role Reversal***]. She'd often be upset, crying, saying my dad was the one and she would never get over it and I'd have to comfort her. But it was weird for me, because my dad left the both of us so I had my own feelings about it, but felt if I shared them it would only upset her more. She had a lot on her plate already, there wasn't necessarily room for my problems [***Coding: Unable to turn to mother for her own needs of support, must provide support instead- AS Role Reversal***].

I see, now can you think of a specific example for why you chose trust to describe the relationship?

I guess like I said, she'd confide a lot in me about her relationship problems or even her stresses with money and work. I guess I remember when she had that boyfriend when I was around 9 years old, and he was kind of a low life, she came to me asking what she should do, should she leave? And I just said, yeah, go for it. But she wouldn't do it, eventually he left her and she was in pieces again [***Coding: Inappropriate confiding of relationship problems- Child put in the role of offering advice. AS Role Reversal***].

3d. Okay, and the fourth adjective you used to describe the relationship from 5 to 12 years old was care. Can you explain why?

I chose care...because through it all she really cared for me. She cleaned my clothes, cooked my meals, walked me to the school bus stop, everything. She worked hard so that we always

had a home. We moved a lot because we were often late on rent but we were never on the street [***Coding: Instrumental support from mother]***.

Great, and can you think of a specific example that demonstrates that? That the relationship had care?

Yeah, sure. Um, one time, when I was about 11, I broke my arm rollerblading. She took me to the nurse next door and we figured out we had to go the hospital. She was in tears, she was so upset that I had gotten hurt, but I was gonna be fine, ya know. It happens to kids all the time. I told her mom, I'm gonna be ok. Calm down! (laughs) [***Coding: Instrumental support from mother]***.

3e. Okay, and the last word you found was grown up. Can you tell me why that describes the relationship early on with your mom?

Hm, I guess because I was always there for her, and had to think about practical stuff like bills and rent, I didn't always feel like a kid. People always said I was very mature for my age and I think that's why I was able to be there for her and give her that support [***Coding: Feeling like an adult and not a kid, for assuming parental responsibilities- AS Role Reversal]***. I felt that she really trusted in me and that's why she could be honest with me about what was really going on, so I really felt like a grown up a lot of the time [***Coding: Praised for being mature, earning trust of mother for taking on this role- AS Role Reversal]***.

Can you think of a specific time or event that shows how the relationship was grown up?

...I remember one time, I had started thinking about why dad didn't help us out a bit with money. We never saw him but mom would talk with him on the phone sometimes [***Coding: AS Role Reversal***]. And I had the maturity to think about hey, we should ask him to pitch in. So I decided the bring up to mom and convince her that she would put her foot down and ask him. Make her see that it was the right thing to do [***Coding: Takes on difficult responsibility, 'parenting the mother'***]. But when I sat with her to talk about it, she totally lost it. It was the worst talk ever (laughs), she got so upset with me and then we had a fight and I went back to my room crying. She started yelling at me that she was the mom and it wasn't my job to think about this stuff, and hadn't she been doing a good job with him? I get her, but I also thought...you do often need my help, it would be cool if another adult could pitch in, but hey **[AS Role Reversal- expresses some frustration, distrust over the role switch]**.

Q5. And okay, I think you mentioned the role your dad played, but could you tell me which caregiver you felt closer to, your mom or your dad, growing up?

Oh, definitely my mom. My dad simply wasn't around. He wasn't really a dad to me, I don't have many memories of him at all.

Q6. Now, when you were upset as a child, what would you do?

Upset? Oh, I'd probably cry or just stew in my room [***Coding: No proximity seeking***].

Q6a. Okay, yeah. And could you tell me, thinking again of those early years between 5 and 12 years old, if you remember what you would do when you were emotionally upset? Feeling sad or angry?

Emotionally? Hm… honestly, I'd often just go straight to my room and write in my diary or cry in bed [***Coding: No proximity seeking***]. If I was really upset, I'd usually try to keep it to

myself, because I knew she had enough to deal with. If I did tell my mom, she'd get worried and then I'd end up comforting her, so… I learned it was better to just handle it on my own ***[Coding: Child's bid support may upset the parent and end up with parent soliciting support from the child - AS Role Reversal]***.

Q6b. Hmm, and can you remember what would happen when you were hurt physically when you were younger?

Same, I'd probably just cry and feel sorry for myself.

Can you think of a time that happened?

I guess if it was really bad, I'd go to mom, like the time I broke my arm ***[Coding: Proximity seeking]*** . But if it was avoidable, I'd keep it to myself because I didn't think mom would really make it better ***[Coding: No Proximity seeking. Avoids proximity for physical pain if possible, mother unable to provide support]***

Q6c. Okay, and do you remember what would happen you were sick as a child?

Ah yeah, um, my mom, for some reason, does not like it when somebody else is sick in the house (laughs) and she's not, like I don't know she likes to get a little bit of attention from that she's a little weird in those circumstances, so she always like, if I was sick with anything, she said she had it too no matter what it was (laughs). So we'd often end up both staying home and watching VHS tapes in bed and making each other instant soup ***[Coding: When child is sick, mother appears to feign sickness as well to get attention. Taking care of each other in the end. Maybe enmeshed theme here as well, but in line with AS Role Reversal]***.

Do you remember any specific times that happened?

No, that was just in general, anytime I was sick we'd be kind of off for the day together, I'd miss school and she'd miss work cuz I guess she came down with whatever I had.

Q6d. Okay. And next, do you remember being held by your mom?

Yeah, for sure, she was very cuddly, always pulling me in for a hug. Definitely when I was sick she liked to hold me. And I'd often give her a lot of hugs too or play with her hair when she was feeling down **[*Coding: AS Role Reversal, calling back to taking care of mom in low emotional moments*]**.
