# Supplementary material for: Organizing adult attachment in alternative ways: A qualitative assessment of schemas antithetical to the secure base script
Source: PLoS One. 2026 May 26;21(5):e0349710. doi: 10.1371/journal.pone.0349710 (PMC13210227; doi:10.1371/journal.pone.0349710)
Supplement: S2 File — (PDF) [file pone.0349710.s002.pdf]

S2: Mock Adult Attachment Interview - Harsh and Threatening Parenting Alternative Schema

To increase the reader's understanding of the coding process, we provide this **mock, fictionalized** Adult Attachment Interview (Q1-Q6), with assigned codes for interview chunks describing potential alternative schemas.

SUMMARY CODING: *Recurring themes of Harsh and Threatening Parenting Alternative schema. Participant descriptives feelings of constant fear and anticipating of targeted, angry outburst from father. Majority of responses from Q3 refer to mother's attempts to protect from fathers' anger (which often fail). One instance of Dismissing/Unresponsive schema, but not recurring thematically throughout interview. Overall, interview reflects the Harsh and Threatening schema throughout each interview section.*

Q1. Could you tell me a little bit about your family when you were growing up and who would you say raised you?

Um, mostly my mom raised me. My dad was there until I was about 14, but I wouldn't say he "raised" me in the way most people think about it. He lived in the same house, but the relationship wasn't… nurturing. It was more like I was constantly avoiding him. I learned really young that if I could keep quiet and out of sight, things would go more smoothly. I used to tiptoe around the house, literally walking on eggshells, because if I got his attention, it usually meant I was about to be yelled at, threatened, or made to feel stupid***[Coding: Walking on eggshells, fearful of father. Maybe AS Harsh and Threatening Parenting]***. My mom was the one who handled most of the day- to- day things - meals, school stuff, making sure I had clothes ***[Coding: Instrumental support Mother]***- but I knew she was also afraid of dad in the house, so she wouldn't say much to defend me when he got angry. So I guess you could say both of us were walking on eggshells around him, just trying to get through each

day without setting him off ***[Coding: Father looming presence in the home, mother relationship also affected. AS Harsh and Threatening]***.

Q2. Now I'd like you to try to describe your relationships with your parents growing up. What was it like with your mom and with your dad between ages 5 and 12?

With my mom, it was… safer. She was a very quiet, reserved woman. She could be stressed and sometimes distracted, but she didn't scream in my face **[*Coding: Contrasting to Dad's behaviour? AS Harsh and Threatening]***. She tried to protect me a little bit from my dad, though she didn't always do that.With my dad, it was tense all the time. He was unpredictable. Some days he'd barely notice me, which was actually better for me. Other days, he'd just erupt - over the smallest things. I remember being maybe 7, I dropped a pencil at the dinner table, and he slammed his hand down so hard the plates rattled. He started shouting about how careless I was and how I "better learn before it's too late." I froze, just staring at my plate, thinking, I should've been quieter. We never had a relationship where I could talk to him or go to him for anything. It was more about scanning his mood the second he walked in the door. I got good at reading the sound of his footsteps, the way he closed the car door, even the speed he walked down the hallway. All of it told me whether I should stay in my room or risk going to the kitchen ***[Coding: AS Harsh and Threatening. Verbally abusive, very unpredictable, Mom attempts to protect from him]***.

Q3. Okay, thank you for describing that. Now, could you give me five adjectives that reflect your relationship with your mother during childhood, between the ages of 5 and 12? I'll write them down and we'll talk about what made you choose each one.

Let's see... Protective. Caring. Tired. Anxious.... and maybe limited.

Q3a. So, for the first word you used to describe that early relationship with your mom, protective, can you tell me a little bit more about that?

Protective because she really did try to keep me out of my dad's way when she could tell he was in one of his moods. I remember her stepping between us in the kitchen, kind of blocking his path toward me when he started yelling. Sometimes she'd make up excuses for why I had to go to my room or why I needed to run an "errand" in the yard. It didn't always work - he could still shout through the walls - but I knew she was trying ***[Coding: Mother trying to act as buffer, but description again of fathers abuse. AS Harsh and Threatening]***.

Can you tell me a specific time when you felt the relationship with your mom was protective?

Yeah, one night I spilled water on the table during dinner, and Dad started to stand up fast, pushing his chair back. She put her hand on his arm and said, "I've got it, sit down." She wiped it up quickly and told me to go get the mop from the garage. That gave me a reason to get out of the room. I could hear him muttering, but he didn't follow me that time ***[Coding: Mother successfully buffers between father's eruption in this case, but again referring to the relationship with father. AS Harsh and Threatening]***.

Q3b. The next word you chose was caring. Can you tell me about that?

Well, I think she was really caring with me and my siblings because she basically took care of everything. All the essentials - food, clothes, making sure I got to school. Maybe, it wasn't the kind of relationship where we'd talk about feelings or do a lot together for fun. It was more like she was managing the logistics of the house, and keeping me fed and clothed was part of that, but it was a lot of hard work because dad was no help in that arena. It showed she cared in her own way **[*Coding: Instrumental support]***.

Can you think of a specific example of the relationship being caring?

Not really specific, just everyday she'd cook for us, keep the house clean, remind us to brush our teeth, that kind of thing **[*Coding: Instrumental support]*.

Q3c. The next word you used was tired. Can you tell me about that?

Tired because she always looked worn down. I think a lot of her energy went into anticipating Dad's mood and trying to manage it. That left her drained **[*Coding: Reference to dads overpowering presence, mother depleted from managing it. AS Harsh and Threatening]*.

Do you have a specific example?

I remember wanting to show her a drawing I made at school, but she was sitting at the kitchen table with her head in her hands, saying, "Not now, I just need a minute." I know now it's not really a big deal, but when you're a little kid...it kinda hurt my feelings ***[Coding: Sign of Dismissing/Unresponsive AS, dismisses child's bid to share artwork and hurts feelings. Does the AS recur?]***.

Q3d. I see. You also said anxious. Why that word?

Because I could see her watching him, the way I did. Her eyes would flick toward the door when she heard his car, and her voice would get quieter if he was nearby. That made me anxious too, because if she was worried, I knew I should be worried [***Coding: AS Harsh and Threatening. Father a source of fear in the home]***. When he wasn't around she was a lot more loose and relaxed and, uh, had more energy to play with us.

Do you have a specific example?

Once, we were baking cookies together, laughing a lot and she suddenly told me to "keep my voice down". She'd heard his footsteps upstairs and didn't want him to get curious about what we were doing **[*Coding: AS Harsh and Threatening*]**.

Q3e. Finally, you chose limited. Can you explain why you chose that word?

Limited because even though she was the safer parent, she couldn't really make things safe. She tried to shield me, but if he wanted to yell or threaten, there wasn't much she could do. I appreciated her efforts, but I knew there was a limit to how much she could protect me. Really, it only made me more angry at my dad for the situation **[*Coding: AS Harsh and Threatening. Mention of anger at the father*]**.

Can you think of a time that shows how the relationship was limited?

Yeah, I was about nine and he was shouting at me for forgetting to turn off a light or something. She stepped in, saying it was her fault, that she'd asked me to leave it on while she looked for something. He ignored her and kept going at me. She stayed in the room, but she couldn't calm him **[*Coding: AS Harsh and Threatening. Father consistently overreacting to small issues*]**.

Q4. Okay, thank you for sharing. Now, let's try this again, finding adjectives with your dad. Could you give me five adjectives that reflect your relationship with your father during childhood, between the ages of 5 and 12?

Yeah, that's easy. Fearful. Unpredictable. Tense. Small. Careful.

Q4a. The first one you said was fearful. Can you tell me a little bit more about that?

Fearful because I never knew what would set him off. Something tiny could trigger a huge reaction. It was always like that [***Coding: Unpredictable, huge reaction. AS Harsh and Threatening]***.

Okay, could you think of a specific time where the relationship felt that way?

Hm, I remember one Saturday morning, I forgot to close a cupboard door after getting cereal. He came in, saw it open, and his whole face changed - his jaw clenched, and his voice went into that low tone. He said, "If you can't respect this house, you don't belong in it," and then started going in on how lazy and useless I was. I was maybe eight. It wasn't just that he yelled - it was the way he'd lean in right up at my face, the look in his eyes. I was always really anxious in the house [***Coding: AS Harsh and Threatening]***.

Q4b. You also said "unpredictable." Can you give an example of why the relationship from ages 5 to 12 felt that way?

Yeah... the unpredictability was constant. One evening, he came home in a good mood, even joked with me about my homework. Ten minutes later, I coughed without covering my mouth, and he just lost it - yelling about "manners" and "disgusting habits," telling me I was going to grow up with "no self- control." The shift was so fast, out of nowhere... That's why I tiptoed everywhere. It wasn't worth the risk of catching his attention if I didn't know what version of him I was going to get [***Coding: AS Harsh and Threatening]***.

Q4c. You mentioned "tense." Can you explain why that word?

Because my whole body would tense up the second I heard his car in the driveway. I'd stop whatever I was doing and go over the mental checklist...Was my backpack put away? Were the dishes done? Was the TV off? I was basically bracing for impact every day. Even when he

wasn't mad, I was on edge, because the next outburst could come at any time**[*Coding: AS Harsh and Threatening. Anticipating reactions to avoid father's anger*]**.

Q4d. The fourth adjective you gave was "small." Can you tell me about that?

Small because nothing I said seemed to matter. If I tried to contribute to a conversation, he'd talk over me or tell me I was wrong without even listening. I never felt like an equal member of the family...more like someone who had to earn the right to be in the room **[*Coding: AS Harsh and Threatening. Makes participant feel small*]**.

Q4e. Finally, you said "careful." Why that word?

Careful because every movement and word had to be calculated. I learned to walk softly, to keep my voice low, to move quickly in and out of shared spaces. I timed my trips to the kitchen for when I heard him in another room. I became an expert in avoiding attention **[*Coding: AS Harsh and Threatening. Walking on eggshells*]**.

Q5. Okay, thank you. Now, can you tell me which caregiver did you feel closer to growing up and why?

Definitely my mom. She wasn't always able to step in, but she didn't try to scare me or anything ***[Coding: Mother-focused response are nearly always contrasted to dad's fearsome behavior. AS Harsh and Threatening]***. With Dad, closeness wasn't even an option. Mom took care of me, and at least we were on the same team.

Q6. When you were upset as a child, what would you do?

If I was upset, I'd hide it [*Coding: No Proximity Seeking]*. Crying in front of Dad could make things worse. He might mock me or say, "I'll give you something to cry about." ***[Coding: AS Harsh and Threatening]***

Q6a. What would happen when you were emotionally upset?

So I'd go to my room, shut the door, and cry quietly into my pillow. Sometimes I'd write in a notebook instead, just to get the feelings out without making noise**[*Coding: No Proximity Seeking*]**.

Q6b. What would happen when you were physically hurt?

If it was small, I'd just deal with it myself. If it was bad, I'd wait until he was out of the house before telling Mom. I didn't want him to see any sign of weakness - it felt dangerous ***[Coding: AS Harsh and Threatening. Dad not a source of support, fear of his reaction]***.

Q6c. Sick as a child?

When I was sick, I tried to keep it low-key. If Dad noticed, he'd accuse me of faking to skip school and make me go. Mom would check on me, but only if he wasn't around making comments about how "kids these days are soft." **[*Coding: AS Harsh and Threatening. Insulting the child for being sick]***

Q6d . Do you remember being held by your father?

No, not once. There was no physical comfort from him - not even a hand on the shoulder in a kind way. If he touched me, it was to shove me out of the way or point me toward something I'd done wrong**[*Coding: AS Harsh and Threatening*]**.
