# Supplementary material for: Organizing adult attachment in alternative ways: A qualitative assessment of schemas antithetical to the secure base script
Source: PLoS One. 2026 May 26;21(5):e0349710. doi: 10.1371/journal.pone.0349710 (PMC13210227; doi:10.1371/journal.pone.0349710)
Supplement: S3 File — (PDF) [file pone.0349710.s003.pdf]

S3: Alternative Schema Coding Guidelines with Novel and Amended themes

This first section includes the amended and novel themes for alternative schemas, with new excerpts from real ASPAN-CAT interviews.

CAREGIVER SOURCE OF DISTRESS

The caregiver's behaviour which elicits general distress, frustration, or anxiety. For example, a chaotic parent who struggles to manage the home may bring anxiety to the child is describes. The child may describe feelings of annoyance, dislike or distrust toward their parent, possibly due to a lack of predictability. There may be general unease when the caregiver is home, even though the child is not directly threatened by the parent's behaviours. Although the adult, in retrospect, appears to have suffered from the consequences of the parent's frustrating behaviours, these caregiving behaviours did not directly target the child and are not described as fear-provoking.

> "*That's similar to my feeling that when he wasn't there that everything went much easier. (mmm) - And yes, that when she came home things were just worse. We had a lot of fights in the house, everyone arguing with each other. And if she wasn't there then we didn't have that.*"

> *"Then when he was going mad, uh, I was just confused who he was and how I should approach him. Um, and also which piece of him who how I should behave. Because*

there was always piece that no matter what I did, when he was in that kind of crazy mood, he was never satisfied with us, so. That was kind of confusing as a kid."

HARSH AND THREATENING PARENTING

The interviewee expresses consistent feelings of a direct "attack" or threat by the caregiver through fear-provoking behaviours. A lack of predictability is described where the child is the direct target of the intrusive or volatile behaviour from the caregiver. Any form of recurrent abuse (sexual, verbal and/or physical) would fall under this schema.

> *"And she started yelling. Just, "Blah-ah-ah-ah. I oughtta 'F' you up!" You know? And I was, like, I just busted out cryin'. You know? It, it really hurt me. You know? And, -- just been real strict in a way. You know? And, the things she used to say sometimes were un, uncalled for. You know"*
>
> *"Well okay, yeah, my aunt she was a um…. um… a, I'll sa, a molester, a sexual molester. And, my aunt had fondled with me, plenty of times… uh, actually had sex with me when I was a little boy."*
>
> *"I bet I was about [Age], and he says well I never wanted you anyway, and that that really hurts, (um-hum) that really hurt"*

Recurring references to physical "punishment" that are extreme considering culture and time period also point to a Harsh and Threatening Parenting schema.

> *"I always got a whoopin' for every single thing that I did. Everything that I did, I got a whoopin' for. I got a whoopin' for, you know, um, -- just sayin' the wrong thing or, um, - -of course, you know, cuttin' up in school, -- uh, not doin' the chores that I was supposed to do."*

"I didn't get talked to, I got whooped, really bad."

Consistent with the original guidelines from Waters & Facompré, the interviewee may express having needed to constantly anticipate the caregiver's mood and "walk on eggshells" around them. However, this differs from the Caregiver Source of Distress schema in presenting a distinct fear of an unpredictable yet unavoidable outburst of anger from the caregiver.

"I was always really afraid to say anything around her, I more or less walked on egg shells around my mother."

"I think that's how my grandfather and I really were, just walked around him very lightly, (um-hum) didn't want to do anything to set his temper off."

SELF-INVOLVED/NEGLECT

The caregiver is unconcerned with the child's daily life experiences, does not play with the child, or puts no effort into their relationship. The child perceived their caregiver as uninterested in spending time together.

"Because I knew he w, he was comin', like, on [Holiday] or birthdays, [Holiday 2}. You know? 'Cause the only thing he would, he would do was come pick us up and take us to his sister's house. Drop us off, or whatever. I mean, that, that was fine, but I wanted to spend time with him. You know? And, I, I, I thought that he didn't wanna spend time with me so I didn't mention it."

"Just passing by. And I'm... like if I was outside, I would wave. And sometimes he would stop and sometimes, you know, he would just wave and keep going?' So I felt like he... he would never make a special trip just to see me. And then when I would see

him, it was always...always these promises. 'I promise I'ma take you to the movies! I'ma take you here. I'ma git you this for your recital, for [Holiday].' And so I wouldn't even ask him that when I saw'em. He would volunteer all these promises. And, of course, nothing, nothing, nothing, nothing"

In some cases, parents' perceived self-involvement may reach the level of neglect, when even basic instrumental care (hygiene, food, cleanliness, and safety of home, life threatening situations, etc.) is not provided by the caregiver.

> *"With my moth, my um, my relationship with my father, it was pretty well. Um, only thing that I disagreed with.. my father, he was on drugs and alcohol real bad. And things that we needed, that we really needed, we didn't have. Because he would mess the money up and stuff. I used to have to go to school, they'd be talking about me, I'm dirty and all this."*

Additionally, we have specified two sub-categories of self-involved schemas, Addiction and Romantic Relationship.

ADDICTION: The caregiver engages in substance or alcohol abuse and cannot meet the instrumental or emotional needs of the child.

> "'Cuz it was a drug..it was addict, it was about drugs all the time, you know. It was about, "Give me some of your dope, or your caine or go cop for me, 'cuz I'm not feeling good", or, it was always about drugs so our relationship wasn't good at all."

ROMANTIC RELATIONSHIP: The caregiver prioritizes their romantic relationships over the child.

"She put her men before her kids. And when I say, "irresponsible," she would just say, 'make sure he ate, you know, before we did?' And that's, you know, really...really irresponsible and inconsiderate, [...]. I mean it was a hurting feelings. Me and my sibling, you know, we ate but it was like he got to eat first? You know? And it shoulda been the other way around or it was always her men first! It was never us, so."

FAVORITISM

This theme encompasses descriptions of a caregiver's consistent preferential behaviour towards a particular or towards other siblings. The child perceives that one or more siblings can do no wrong in the parent's eyes. Perhaps one child is seen as the "golden child" in the family, leaving the participant with a feeling that they in turn lacked attention and care.

"He used to always make me mad because he always took my little sister around with her whenever he was going to the grocery store, or going shopping for clothes, or whatever. And what made me so mad is because he used to sneak and do it. (Okay.) So maybe he'll wake her up early and they'll just leave. By the time I'm up, they'll be gone. And then, she used to always come back with like little things and something that he bought. He'll buy her things and whatever because she went. That's why I always used to like to go with him, but....for some reason... He...he used to say, it's because he didn't want to comb my hair. It took too much time or whatever, but I used to...sometimes, I used to cry, and sit in the window, and wait for them to come back."

SCAPEGOAT: Alternatively, the participant may also self-identify as the "problem child". They may also be used as a "Scapegoat", where undue blame is placed upon this child and never other family members. The subtheme describes the child-caregiver relationship as being framed in the caregiver's contrasting treatment between siblings, but differs in that

overwhelmingly negative attention was placed on the participant. The child may self-identify as the "problem child". For example,

> *"Shouting at me......anything happened in the house it was my fault ... if the dishwasher broke it was me, not one of the boys…"*

RESTRICTIVE

The caregiver restricts or discourages child from exploration, play, activities and/ or self-expression. In doing so, they are hindering the support exploration aspect of the secure base script. The participant may express a certain frustration, feeling stifled, a lack of freedom to be themselves or to engage in age- appropriate activities (i.e., play outside, sleepovers, after-school programs) or pursue their own interests. The child is not encouraged and may even be discouraged in their own personal development.

> *"It was like a church camp thing too, I mean it was like, couldn't be more, uh, safe or well taken care of and well looked after and all that, and she told me that, yes, you can go. And the day I was supposed to leave she said, no, the weather doesn't look very good or, I don't want you to h you know with all those kids you're going to catch cold, you're going to get sick, that was a big thing with her, you're gonna get sick, so she said no and I made this big, you know, stink about it, and, right up until, I think it was, I was even late, it was even past the time when I was supposed to be where the bus was going to take us, and my dad said, you know, Person 3, you know, let him go, and so, and they kept arguing and I went and I grabbed my stuff and I just headed out the door and I ran, I got to the bus on time and went and that was, and that was it."*

INCOMPETENT

The participant calls into question the competence of the attachment figure as a caregiver, to the point that the caregiver's parenting abilities are consistently called into question or seen as outright lacking. An Incompetent alternative schema may be present where there are multiple AAI_{sbs} codes assigned for a negative Stronger and Wiser secure base expectation, indicating that the caregiver's authority and competence are brought into question. A negative Stronger and Wiser secure base expectation is coded when the interviewee describes considerable doubt in the caregiver's ability to resolve a conflict (Waters & Facompré, 2021). The participant may see the caregiver as incapable of parenting, despite their efforts, and as undeserving of their respect.

> *"I didn't like her. She .... I guess and my step-mom probably don't know that she did that, but she gave me bad vibes, every since I was little. Because she didn't know a lot, when I was little. And I was learning. I was a very quick learner. I taught my mama to do math, calculate... certain things, me and my daddy. And I would hear my daddy say things to her, you know, and I would use 'em against her. Because I was like very bright and very manipulative, you know. If she said something to me, I'd go tell my daddy, he argued with her, about me. So it, I guess she felt like, "You know this little worthless child". You know, and I felt it! I felt how she felt, you know, so. So that made me, you know, talk crazy to her if I could. And, "Forget you!" And yeah. Stuff like that. And I didn't never get no whipping for it, so ... I never was really punished about it."*

> *"That kind of made my relationship with him, I didn't see him as, you know, Dad anymore, it's like this man who, umm, what's the word…whom I didn't have much respect for, I'll put it that way, I didn't have a whole lot of respect for him after that cause everything."*

The following section includes guidelines for identifying alternative schemas originally written by [ANONYMIZED] with novel, de-identified excerpts taken from the ASPAN-CATS sample.

CHILD PUT IN THE MIDDLE

The child notices parental discord on a consistent basis and/or is asked to play an active role in resolving parents' issues (e.g., forced to choose sides, one parent speaks negatively about the other).

> *"And um… and I used to be, and I was scared to go tell my father. Because I was um, I had this fear that she would hurt my father. And so um, I never told him."*

> *"He blames me because things are bad between him and my mom. And I just blame him for a lot of my problems"*

DISMISSING/UNRESPONSIVE

In response to a clear signal for help, the caregiver decides whether the conflict or challenge warrants the provision of secure base support. If the caregiver feels the child's concerns are valid, intervention is provided; if not, the child is dismissed or (as in the example below) explicitly told to resolve the problem on their own.

> ***"And what would usually happen when you were ill as a child?***

> *Shipped off to Papa's [grandfather]."*

> *"And with girlfriends, couple stuff, then she actually only wants to know if something is serious. And in the meantime, she does not want to hear about it."*

Importantly, the Dismissing/Unresponsive alternative schema is not to be confused with the Dismissing attachment classification in traditional AAI coding.

ENMESHED/COMPANIONSHIP

The attachment relationship is defined by explicit mention that the child serves an important role as a source of comfort or companionship to the parent.

> *"Yes, you could say anything. And she said, I think, a lot which perhaps she shouldn't say to children, but in that sense, you could, you weren't judged on things."*

Although some might argue that the apparent "bond" between the two signifies a healthy attachment relationship, in terms of the secure base concept, this violates the natural order of things. This is because relationships defined by companionship may result in a blurred distinction as to who is the secure base and who should be the recipient of support. Consequently, these kinds of schematic representations make it less likely that the child will seek support from the caregiver due to the absence of a well-established hierarchy (like one might expect in a peer relationship). Enmeshed characterizations of the relationship can also result in reciprocal or shared inductions of distress owing to the fact that no single figure is effectively resolving the problem (as in the example below).

> *"So I always thought that my aunt was my best friend and I know I shouldn't have been at some of those parties. But sometimes, she would take me and I knew all her friends. And, of course, I was the only child there. So I'm thinking I'm a grown-up, but I'm really a child."*

ROLE REVERSAL

Unlike the Enmeshed/Companionship alternative schema, the child partly or completely takes on the role of the parent. As illustrated in the example below, we find that trouble usually follows when the parent later tries to act as a secure base.

> *"Um, um not, not so much specifics I can't unfortunately but um, on both parts it was because, I would, I, felt like semi responsible for her because she, she can see but she's partially deaf, so I had to help a lot to get her get her around, make sure she didn't step into traffic or something like that."*

> *"I didn't feel like a child should feel I guess you could say, to a mother, so- it was very distant, it was like, our relationship was more or less like you're an adult, I'm an adult, but you're a child when I say you're a child kind of."*

PARENT DEMANDS EXCELLENCE/STRIVING FOR ACCEPTANCE

The parent–child relationship is defined by a superficial expression of fondness, in which the child is displayed as a trophy or accessory to parents' accomplishments. As a result, the child comes to internalize his or her own successes (i.e., achievement in academics or sport) as being fundamentally linked to their parents' affection. The child with this schema consistently tries to please the parents or live up to their standards, though affirmations that the child has succeeded in this rarely, if ever, appear.

> *"Uh it was very hard to please her because he is v, she is so type A personality and just extremely high standards and uh lots and lots of rules and I could never keep track of all the rules so I was always felt like I was in trouble. And uh so that was really hard and my dad -wasn't really disciplining in the same way."*

"I was the runner-up for the swimming competition, (Um-hmm) and, um, they gave the, the first place winner, they gave a trophy, and I didn't get anything for being the runner-up, (Um-hmm) but, so my grandma went out and bought a trophy for me, and my dad was mad about that, and I mean these words just affect so much of me today, but he said, "You shouldn't buy yourself trophies for second place." So, second place was not good enough for him."

SUBJUGATED/SUBORDINATE

The parent–child relationship is defined by a subordinate role, centred on what is expected of the child (i.e., duties and schedules) rather than on what can be expected from the parent as a secure base. Expectations to this effect often describe deep-rooted feelings of worthlessness, helplessness, and inequality.

"Here I am, you know, doing all these local delivery jobs, and watching my brothers, and cooking food at home."

"My mother always, um, wanted me to be around the house. You know, it was not - I couldn't -well, I think I had a lot of chores to do around the house, around the property basically. So, you know, she was basically strict, you know, get things done. And that what she wanted to be done, you know, clean the house. You know, it had to be done or get a beatin' or whatever. You know, uh, so that -that was the strictness. And then school work 'cause she was a [Occupation 1]. And basically, you know, she was strict on me because I was supposed to be settin' an example for my brother, so - so that was -that was 'cause of that."

TIT-FOR-TAT

In clear contrast to the “Subjugated/Subordinate” schema, the parent and child both find themselves in equal and competing roles. This can often lead to contentious encounters as might be reflective of sibling relationships.

> “Um, it was always confusion, ‘cuz we was always, when I got a little older I was, going off on him, he was going off on me.”

> “No, I wasn't never scared of her. Never worried about her or nothing. Scared if she snapped, flip out on me or something. But that, that was it. I used to know when she would break something or get mad, I’d be knowing. I’d be push, trying to push my limits, push her boundaries.”
