# Supplementary material for: Organizing adult attachment in alternative ways: A qualitative assessment of schemas antithetical to the secure base script
Source: PLoS One. 2026 May 26;21(5):e0349710. doi: 10.1371/journal.pone.0349710 (PMC13210227; doi:10.1371/journal.pone.0349710)
Supplement: Attachment — Submitted filename: Response to Reviewers_Alt Schema Study.docx [file pone.0349710.s005.docx]

Response to reviewers

We thank the academic editor and the two anonymous reviewers for their time and guidance following the first review of our paper titled "*Organizing Adult Attachment in Alternative Ways: A Qualitative Assessment of Schemas Antithetical to the Secure Base Script*." We found their comments helpful and hope they will agree that the paper has been improved. We have addressed each of the reviewers’ comments as follows.

[In response to Reviewer 1	2](#_Toc227327480)

[*1.*	*On the composition of the dataset across linguistic and national samples*	2](#_Toc227327481)

[In response to Reviewer 2	3](#_Toc227327482)

[1. On expanding on the role of alternative schemas and their importance within attachment theory in the Study Aims	3](#_Toc227327483)

[2. On providing further details on sample characteristics	5](#_Toc227327484)

[3. On the implications of alternative schemas for attachment insecurities and attachment styles	5](#_Toc227327485)

[4.	On theoretical implications of alternative schemas in relation to cultural norms	7](#_Toc227327486)

[5.	On clearly defined limitations section addressing gender, culture, and methodology	8](#_Toc227327487)

In response to Reviewer 1

## ***1. On the composition of the dataset across linguistic and national samples***

*‘The dataset includes transcripts in three different languages (English, Dutch, and French). It would be helpful for readers if the authors provided more information about the composition of the dataset. Specifically, what proportion of the analyzed transcripts came from each linguistic or national sample? And were there any notable differences between these subsamples in terms of the identified schemas or thematic patterns?*

*While such distinctions may not be central to the qualitative aims of the study, they are nevertheless informative for understanding the scope and diversity of the analyzed material. Additionally, it would be interesting to know whether any attachment-related schemas or themes appeared exclusively or predominantly in one linguistic or cultural context but not in others. Even a brief note addressing this issue would enrich the interpretation of the findings.’*

We thank the reviewer for the encouraging remarks and thoughtful feedback. We have added the following breakdown of transcripts by language and country of origin, as well as additional demographic information, to the *Participants* section, page 12 (lines 239 to 253). The section reads:

“As part of the ASPAN network, AAI transcripts were included from English (62%), Dutch (28%), and French-speaking (8%) samples. Approximately half of the Dutch and French transcripts were translated into English by a professional translation service. Transcripts were collected from Canada (8%), the Netherlands (27%), the United Kingdom (5%), and the United States (60%). All interviewees were either parents or expecting a child, with 93% of the total sample female and, on average, 28 years old. A total of 1,592 AAI transcripts collected within ASPAN-CATS were coded for secure base script knowledge by our team of seven trained coders, drawn from both normative (38%) and high-risk populations (62%). High-risk status was defined across contributing studies by the presence of one or more risk factors, including adolescent parenthood, parent psychopathology, parental substance abuse, parental history of abuse or trauma, and demographic risk indicators such as poverty. In terms of ethnicity, 63% of participants identified as Caucasian, 30% as African American, 3% as Hispanic, 1% as Asian, and 2% as other, with 13% reporting immigrant status. Roughly 50% of participants had completed tertiary education.”

Regarding schema differences across linguistic or national subsamples, systematic comparison was beyond the scope of the current study. As a qualitative thematic analysis, this study aimed to identify and characterize the content of alternative schemas in a large, diverse sample, rather than to compare their prevalence or distribution across subgroups. We acknowledge this as a valuable question, however, and note that this is precisely the kind of question the first author intends to pursue in upcoming quantitative work, examining schema prevalence and content across national samples. We have added a brief reference to linguistic diversity in the existing passage in the *Reaching Saturation* section of the Discussion, page 26 (lines 556-567), which already addresses the broader question of cultural scope and invites future research on this point (additions are underlined). The section reads:

“We reached a saturation of themes for alternative schemas during coding of this large corpus of AAIs, further increasing confidence in the schemas identified. After a period with no additional themes emerging beyond Favoritism, Restrictive, and Incompetent, the coding team conducted a saturation check at 814 transcripts coded. No evidence for additional novel themes emerged beyond this point. Although this may indicate that the sample was sufficiently diverse to cover the variation present in Western, English-dominant samples, novel themes may occur in other linguistic contexts or cultural populations. The attachment field has been widely criticized for its assumptions of universality in attachment (i.e., Behrens, 2016; Thompson, 2019). Indeed, it is possible that certain elements assumed to be central to secure base provision in Western society may not necessarily be present in other cultures.”

# **In response to Reviewer 2**

1. On expanding on the role of alternative schemas and their importance within attachment theory in the Study Aims

*‘The aim of the study could be expanded to include the role of alternative schemas. I suggest that the authors more explicitly highlight the importance of alternative schemas within attachment theory.’*

In response to Reviewer 2's feedback, we have expanded on the theoretical significance of alternative schemas and their place within the broader attachment framework. We have added the following paragraph to the theoretical section on *Scripts and Schemas* on pages 8 and 9 (lines 185-199) that further underscores the potential impact of differentiating among the alternative schemas:

“Empirical work examining the antecedents and outcomes of alternative schemas has yet to follow. Measured using the ASA, secure base script knowledge has been linked to the quality of caregiving received in childhood and to later adult depression symptoms and romantic relationship quality (e.g., Dagan et al., 2021; Steele et al., 2014), underscoring how early caregiving experiences may become encoded in representational scripts that carry consequences across the lifespan. Emerging work examining the impact of maternal childhood abuse versus neglect on infant brain development further suggests that different types of adverse caregiving experiences may have distinct developmental consequences (Lyons-Ruth et al., 2023). This raises the question of whether such experiences also give rise to qualitatively different representational schemas in the adult mind, as captured in the AAI. Whether the perceived relational dynamics captured by alternative schemas, rather than broader classifications, carry differential relevance for such outcomes remains an open and important question. Before such empirical questions can be addressed, a necessary first step is to systematically characterize and refine the set of alternative schemas amongst large, risk-diverse samples.”

We have also added some text to the ‘Current Study’ paragraph on page 10 (lines 205-212) to clarify the role of alternative schemas and strengthen our overall aim (additions are underlined):

“In trying to understand how individuals make meaning of childhood experiences with intrusive or distressing caregivers, it is important to consider that the absence of secure base expectations may not simply indicate a lack of representational structure, but may also reflect the presence of alternative ways of organizing expectations about attachment relationships. The purpose of the current study was to explore the content and scope of alternative schemas in the AAI, as a step towards understanding how characterizations of unpredictable and distressing caregiving in childhood may become internalized as cognitive blueprints in adulthood. We therefore conducted a systematic, semi-open assessment of the preliminary set of alternative schemas within a large, risk-diverse set of 14 samples that are part of the Attachment Secondary Processing and Analysis Network (ASPAN). We first evaluated whether the set of alternative schemas identified by Waters and Facompré (2021) also occurred in the current dataset in the form originally described. Second, we examined whether novel themes could be identified, justifying the addition of novel alternative schemas to the set. This broader set of alternative schemas might contribute to further nuancing our understanding of attachment representations, particularly in individuals who experienced adverse caregiving environments.”

2. On providing further details on sample characteristics

*‘The sample is not sufficiently defined or described. I would appreciate it if the authors provided more detailed sample characteristics, including relevant demographic information.’*

We have included additional sample characteristics and demographic characteristics on page 12 (lines 239-253). Please see our Response to Reviewer 1, comment 1.

3. On the implications of alternative schemas for attachment insecurities and attachment styles

*“In the discussion section, I recommend elaborating on the theoretical meaning of these alternative schemas in the context of attachment insecurities. Specifically, the implications for different attachment styles should be clarified. How do these findings contribute to our understanding of internal working models?”*

We have added a new subsection titled *Theoretical meaning of alternative schemas* to the Discussion on pages 28-29 (lines 605-631), in which we elaborate on the theoretical implications of alternative schemas in the context of non-autonomous attachment representations. We note that in line with AAI convention, we use the term ‘non-autonomous’ rather than ‘insecure’ throughout the manuscript, or specify the dismissing or preoccupied representations.

In this new subsection, we position alternative schemas as offering a complementary lens to existing AAI classifications: rather than assessing non-autonomous attachment through discourse incoherence or attentional strategies, alternative schemas capture the explicit content of attachment-relevant experiences as recalled during the AAI. We further elaborate on how mapping alternative schemas onto non-autonomous classifications may help specify which relational expectations and experiences underlie dismissing or preoccupied attachment strategies. The section reads:

“***Theoretical meaning of alternative schemas***

Beyond reliability and coding utility, capturing distinct schemas may carry theoretical meaning in its own right. These schemas reflect a qualitatively distinct representational organization of attachment-relevant experiences with primary caregivers. Rather than simply indicating the absence of secure base script knowledge, they outline in detail the recurring expectations of how caregiving relationships function under conditions of fear, distress, role confusion, unpredictability, or emotional unavailability.

This distinction may offer a complementary approach to understanding non-autonomous attachment classifications. With the move to the level of representation (Main, Kaplan, & Cassidy, 1985), security and insecurity in adult representations of attachment are assessed on the basis of discourse coherence and attentional strategies during the AAI, where dismissing and preoccupied classifications describe ways in which these discourses of attachment-related experiences are limited and distorted. Alternative schemas offer a different lens: rather than how narratives are organized, they capture the explicit content of attachment-relevant experiences as recalled during the AAI. Mapping the alternative schemas onto the non-autonomous classifications may help capture which expectations and behaviors underlie preoccupied or dismissing attachment strategies. For instance, dismissing representations may not only manifest through implicit discourse indicators such as idealization or lack of memory (Hesse, 2008), but also through explicit, temporal-causal, relational content described by schemas such as Self-Involved/Neglect or Incompetent. Similarly, discourse indicators for preoccupied (e.g., passive speech, difficulty moving on from topics) may be further specified through schemas such as Enmeshed or Role Reversal. Examining this additional layer of specificity, detailing the particular parenting behaviors that led to the caregiver being perceived as a source of distress rather than support, may hold relevance for both research and clinical practice. Empirical work directly examining the overlap between alternative schemas and AAI classifications is needed to test these propositions.”

Furthermore, we use the term "representation" rather than "internal working model" throughout the manuscript, as this aligns more closely with the theoretical framework of the secure base script, within which alternative schemas are embedded. Similarly, we situate our findings within the AAI-based classification tradition of attachment representations rather than the self-report attachment styles literature. However, we acknowledge that the question of how alternative schemas relate to romantic relationship quality, among other spheres of adult life, is an important direction for future work. We have expanded the *Antecedents, Consequences, and Utility* section on pages 29 and 30 (lines 643-675) accordingly to address these implications:

“The relevance of such questions is underscored by existing work on secure base script knowledge more broadly. Dagan and colleagues (2021, 2024, 2025) showed that greater secure base script knowledge was associated with better emotional and physical health, as well as higher romantic relationship quality, specifically lower perceived hostility from a romantic partner. Furthermore, secure base script knowledge mediated the relation between maternal and paternal sensitivity in childhood and romantic partner hostility and emotional health. This finding on secure base script knowledge aligns with previous research, supporting the idea that this form of attachment representation, built on repeated experiences of sensitive caregiving (Steele et al., 2014; Nivison et al., 2020; Schoenmaker et al., 2015; Waters et al., 2017), may influence the processing of interpersonal information and behaviours later in life (Collins & Sroufe, 1999; Dykas & Cassidy, 2011; Dagan et al., 2024; Waters et al., 2013, 2018). Considering that alternative schemas similarly reflect expectations based on childhood experiences with primary caregivers, it remains an open question how these schemas, which specify content that conflicts with elements of secure base provision, may also impact adult functioning.

The alternative schemas may offer both research and practical utility by providing detailed information on how adults make sense of caregiving relationships and organize their broader expectations of others. What it means, representationally, to organize expectations around experiences of threat and fear may be vastly different from experiences of taking on the caregiving role: one might expect that an individual describing the Harsh and Threatening Parenting schema perceives the world as a particularly hostile place, in contrast to someone with a schema scored as Enmeshed. Gaining a better understanding of the antecedents and consequences of such cognitive schemas may therefore be a promising avenue for both researchers and clinicians. In this regard, Raby and colleagues (2021) showed that secure base script knowledge improved following intervention in parents referred by Child Protective Services, and that this increase mediated improvements in parental sensitivity seven years later. These findings support the view that cognitive scripts are malleable and can be modified through new experiences (Bowlby, 1988; Schank & Abelson, 1977; Waters et al., 2019; but see Witte et al., 2024, for null effects on script change following intervention, which the authors attribute to possible sleeper effects). The current study offers further description and delineation of alternative schemas in a risk-diverse sample, providing a first step towards future research on whether cognitive scripts centered on themes of fear and unpredictability may also show malleability and openness to change.”

4.	On theoretical implications of alternative schemas in relation to cultural norms

***‘****The manuscript would benefit from a deeper discussion of the theoretical implications of these schemas, particularly in relation to cultural norms.’*

We have expanded the *Reaching Saturation* section of the Discussion on page 26 (lines 568-574) to more explicitly address the role of cultural context in shaping the content of alternative schemas.

Given the qualitative and exploratory nature of the current study and the absence of established reliability for schema subtypes, systematic cross-cultural comparison was beyond the scope of the present study but represents an important avenue for future research. The section reads:

“Beyond sample diversity, the cultural context in which caregiving takes place may shape the specific content of alternative schemas. What constitutes fear, distress, or a failure of secure base provision is partly culturally situated. During coding, for instance, corporal punishment was not automatically coded as Harsh and Threatening Parenting unless the narratives reflected fear, an approach that attempted to account for cultural variation in disciplinary norms. Future work examining schema content across diverse cultural settings would be a valuable direction.”

5.	On clearly defined limitations section addressing gender, culture, and methodology

‘*Finally, I suggest including a clearly defined limitations section that addresses potential issues related to gender, culture, and methodology*.’

We have added a dedicated *Limitations* section to the manuscript on page 30 (lines 677-698). We note that in qualitative research, reflection on methodological considerations such as saturation, reliability, and cultural scope is integral to the analytic process, and these points were therefore addressed within their relevant sections of the manuscript. The new *Limitations* section complements these discussions by bringing the key points together and addressing two considerations not previously discussed: the predominantly female composition of the sample and its implications for generalizability to male caregivers, and the potential influence of AAI translation on the narratives elicited across the three language groups (additions are underlined). The section reads:

“***Limitations***

While the current work provided added detail to the AAIsbs coding schema for identifying alternative schemas, it remains an exploratory study. The interview coding was conducted in the context of discovery, and reliability was not verified for the alternative schema subtypes. While reliability is not a necessary construct for the nature of a qualitative, thematic analysis (but see Van IJzendoorn & Miedema, 2025, for a contrasting perspective), we do remain cautious in the implications of our findings. Before testing the many hypotheses generated above, establishing the psychometric properties of the schemas is an important first step.

Additional features of this study may limit the generalizability of the findings. Although the sample was risk-diverse, it remains Western, and theme saturation may not extend to more geographically diverse populations. The thematic analysis thus does not explicitly account for whether different caregiving norms may give rise to additional or differently nuanced schemas. Furthermore, although the sample included both mothers and fathers, and schema types did not appear to differ by gender on initial inspection, the sample was predominantly female. Findings may therefore not fully capture the range of schemas that could emerge in male caregivers. Finally, although transcripts were collected and coded across three languages, the preliminary schema definitions and coding guidelines were developed in English. This may limit how well subtle cultural or linguistic nuances are captured in childhood narratives. The AAI itself was originally developed in English, and translations may carry subtle differences in how questions are phrased or interpreted, potentially influencing the narratives elicited and, in turn, the schemas identified.”

We hope to have satisfactorily integrated the reviewers’ helpful comments and thank them again for their support in improving our paper.
